# Effects of Using Barbed Suture in Myomectomy on Adhesion Formation and Adverse Pregnancy Outcome

**DOI:** 10.3390/jpm13010092

**Published:** 2022-12-30

**Authors:** Seyeon Won, Su Hyeon Choi, Nara Lee, So Hyun Shim, Mi Kyoung Kim, Mi-La Kim, Yong Wook Jung, Bo Seong Yun, Seok Ju Seong

**Affiliations:** 1 Department of Obstetrics and Gynecology, CHA Gangnam Medical Center, CHA University School of Medicine, Seoul 06135, Republic of Korea; 2 Department of Obstetrics and Gynecology, CHA Ilsan Medical Center, CHA University School of Medicine, Goyang 10414, Republic of Korea

**Keywords:** uterine myomectomy, robotic surgical procedures, laparoscopy, uterine fibroids

## Abstract

Background: There is still concern regarding postoperative adhesion formation and adverse effects on pregnancy outcomes caused by barbed suture (BS) after myomectomy. The aim of this study was to compare the postoperative adhesion and pregnancy outcomes between conventional suture (CS) and BS after minimally invasive myomectomy (MIM) by robotic myomectomy (RM) or laparoscopic myomectomy (LM). Methods: The medical records of 94 women who had undergone MIM with CS and 97 who had undergone MIM with BS and achieved pregnancy were reviewed. Postoperative adhesion was evaluated following cesarean section. Results: The number of removed myomas was greater (5.3 ± 4.6 vs. 3.5 ± 3.1, *p* = 0.001) and the size of the largest myoma was larger (7.0 ± 2.2 vs. 5.8 ± 2.7 cm, *p* = 0.001) in the BS group relative to the CS group. A total of 98.9% of patients in the CS group and 45.4% in the BS group had undergone LM (*p* < 0.001), while the others underwent RM. There was no significant difference in the presence of postoperative adhesion at cesarean section between the BS and CS groups (45.5 vs. 43.7%, *p* = 0.095). Additionally, there were no intergroup differences in pregnancy complications such as preterm labor, placenta previa, accrete or abruption. Note also that in our logistic regression analysis, the suture type (BS or CS) was excluded from the independent risk factors regarding postoperative adhesion formation. Conclusions: Our data indicated that the incidence of postoperative adhesion after MIM with BS was similar when compared with CS. Also it seems that the suture type does not have a significant effect on pregnancy outcomes.

## 1. Introduction

It is known that quickly and properly tying surgical knots with conventional suture (CS) presents a significant challenge in minimally invasive myomectomy (MIM) [1]. Knot-tying, which in some contexts is a physically and mentally stressful process, can be obviated by using barbed suture (BS) instead. Given its barbed surface, BS does not need to be knotted by the physician. In fact, BS has already been proved to be effective in laparoscopic myomectomy (LM), reducing both suture time and, thus, intraoperative blood loss when compared with CS [2,3].

However, the major concern with BS is the risk of barb-induced adhesion formation and inflammation. Several studies have reported bowel obstruction and perforation due to adhesion formation secondary to BS [4,5]. Besides, a randomized controlled trial (RCT) with an animal model has revealed that BS is significantly more associated with adhesion formation than is CS [6]. Contrarily though, another RCT/animal model found no difference in adhesion formation between BS and CS [7]. Likewise, there was no BS/CS difference in a unique prospective study in which postoperative adhesion was evaluated at 2nd look laparoscopy [8]. All of this notwithstanding, it should be noted that adhesion formation and pregnancy outcomes after MIM with BS are not as clear or as well established as they are for MIM with CS.

We have performed MIMs with BS for several years due to its several advantages, but have begun to wonder if BS might actually lead to postoperative adhesion and even adverse pregnancy outcomes. The aim of the present study, then, was to compare postoperative adhesion and pregnancy outcomes between BS and CS after MIM by robotic myomectomy (RM) or LM. Postoperative adhesion was evaluated following cesarean section.

## 2. Materials and Methods

Our retrospective cohort study, with the approval of the relevant institutional review board (GCI-2022-09-006), was performed in a single gynecological surgery center using data collected between September 2015 and December 2020. The medical records of 94 women who had undergone MIM with CS and 97 who had undergone MIM with BS and achieved pregnancy were reviewed. Among them, postoperative adhesions and adverse pregnancy outcomes were evaluated for women who had undergone cesarean section. Moreover, all data were analyzed again only with patients who underwent LM (not RM) to unify surgical platforms.

### 2.1. Surgical Methods

MIM had been performed by either RM or LM. RM had been performed with the da Vinci-Si system (Intuitive Surgical, Inc., Sunnyvale, CA, USA). Four ports, including a 12-mm camera port (at the umbilicus), and three 8-mm side ports, were inserted. After insertion of all of the trocars, the surgical cart was docked vertically. The robotic right arm held monopolar curved scissors, and the left arm held tenaculum forceps. A solution of vasopressin diluted to a 0.25 U/mL concentration was injected directly into the myoma. The monopolar curved scissors performed the incision, while the tenaculum forceps applied counter-traction. LM with four ports was performed by a technique similar to the robotic one. The V-loc™ (Covidien, Dublin, Ireland) for barbed continuous suture or Vicryl (Ethicon, Somerville, NJ, USA) for conventional interrupted suture was employed to suture the uterine wall. Myomas were retrieved by electric-power morcellation.

### 2.2. Statistical Analysis

Student’s *t*-test for comparison of continuous variables and the χ^2^ test for categorical variables were used. For determination of non-parametric statistics, Fisher’s exact test was used. Variables (*p*-value < 0.2) from a univariate analysis were included in a subsequent multivariable logistic regression model. The analyses were performed using SPSS version 24.0 (IBM Inc., Armonk, NY, USA), and *p*-values < 0.05 were considered statistically significant.

## 3. Results

The two groups’ baseline characteristics at myomectomy are provided in Table 1. Older age (35.4 ± 3.2 vs. 32.9 ± 3.2, *p* < 0.001) and higher BMI (22.8 ± 3.5 vs. 21.4 ± 2.8, *p* = 0.003) were shown in the BS group relative to the CS group. The number of removed myomas was greater (5.3 ± 4.6 vs. 3.5 ± 3.1, *p* = 0.001) and the size of the largest myoma was larger (7.0 ± 2.2 vs. 5.8 ± 2.7 cm, *p* = 0.001) in the BS group relative to the CS group. A total of 98.9% of patients in the CS group and 45.4% in the BS group had undergone LM (*p* < 0.001), while the others underwent RM. Heavier tumor weight (198.3 ± 185.7 vs. 127.2 ± 129.8 g, *p* = 0.003) and longer operative time (157.1 ± 59.5 vs. 126.9 ± 44.94 min, *p* < 0.001) were noted in the BS group. Otherwise, there were no significant intergroup differences. When we analyzed it without RM patients, there was no significant differences except older age in BS group (35.6 ± 3.3 vs. 32.9 ± 3.2, *p* < 0.001). The pregnancy outcomes are summarized in Table 2; as is apparent, there were no significant intergroup differences. Table 3 shows the surgical characteristics and outcomes at cesarean section. There were no significant intergroup differences, neither in presence of adhesion (*p* = 0.095) nor in adhesion site (*p* = 0.158). Even when we analyzed it without RM patients, there was no significant differences regarding adhesions except age (*p* = 0.037). Moreover, as shown in Table 4, there were no significant differences in pregnancy complications such as preterm labor, placenta previa, accrete or abruption. There was no fetal anomaly case. Note that in our logistic regression analysis, the suture type (BS or CS) was excluded from the independent risk factors regarding postoperative adhesion formation.

## 4. Discussion

To the best of our knowledge, this is the largest cohort study to evaluate postoperative adhesion and adverse pregnancy outcomes with BS as compared with CS. There have been two studies on postoperative adhesion after myomectomy with BS. In 2020, Kumakiri et al. conducted a prospective comparative study (LM with BS vs. CS) [8]. The postoperative adhesions were evaluated by a 2nd look laparoscopy, and classified and graded. Four patients in the BS group (n = 22) and eight patients in the CS group (n = 22) showed postoperative adhesion (18.2 vs. 36.4%, *p* = 0.31), and it was concluded that the incidence of postoperative adhesion following the use of BS in LM was similar to that following the use of CS. The results of another prospective study by Giampaolino et al. were similar: having performed a postoperative evaluation by office transvaginal hydrolaparoscopy, they observed adhesion in four patients in the CS group (26.7%) and three patients in the BS group (17.6%) (*p* = 0.5) [9]. Our data carry similar implications. The rate of adhesion was not significantly different between the BS and CS groups (43.7 vs. 45.5%, *p* = 0.95). Of course, our study, given its retrospective nature, has an insurmountable limitation in that adhesion was not classified or graded. Nonetheless, we think that two points in our data are worth noting. Firstly, the operative time at cesarean section was not statistically different between the two groups, as indicated in Table 3 If the degree of adhesion was different between the groups, the operative time at cesarean section would have been correspondingly different. Secondly, although the BS group was expected to have more postoperative adhesion due to the removed myomas’ characteristics (i.e., larger in both number and size), adhesion at cesarean section did not, as already noted, significantly differ between the two groups. It is known, based on Takeuchi et al.’s prospective study [10], that the number and diameter of removed myomas are the factors influencing adhesion development. According to Kumakiri et al.’s study, the enucleated subserosal myoma number (odds ratio, 3.29; *p* < 0.001) and largest-myoma diameter (odds ratio, 1.05; *p* < 0.001) were associated significantly with wound protrusion, a critical factor with respect to adhesion [11]. Furthermore, several studies have demonstrated that the length of the myomectomy incision is an important factor in adhesion development [11,12,13,14]. As Table 5 shows, the number and diameter of removed myomas were the risk factors regarding adhesion formation in the present study, too.

The still-limited data on the impact of BS on pregnancy outcome do not allow for any exhaustive analysis or definitive conclusions on the safety of this technique or associated risks of adverse pregnancy outcome [15]. There has been just one relevant retrospective comparison study [16], which reported two cases of intrauterine growth restriction, three cases of preeclampsia and two cases of preterm birth in its CS group (n = 38), and in its BS group (n = 32), one case of placenta previa, two cases of intrauterine growth restriction, two cases of preeclampsia and two cases of preterm birth; consequentially, adverse pregnancy outcome was deemed to be not statistically different between the two groups. Additionally, there is one RCT that compared cellular composition and proliferation in uterine healing between BS and CS in a sheep model [17]; it found that BS afforded a similar uterine healing effect to that of CS. Our own results showed that adverse pregnancy outcomes were not statistically different between BS and CS; however, it will be necessary to investigate the real impact of BS on pregnancy outcomes with a further, large prospective study.

This study has some limitations due to its retrospective nature. First, postoperative adhesions at cesarean section were not systemically classified, and the degree was not assessed. Second, the two groups were not fully equal for best comparison. Particularly, the surgical platforms were not uniform. Although the surgery type (LM vs. RM) was excluded from the independent risk factors regarding postoperative adhesion formation, the fact that most of the CS group (98.9%) had been operated on by LM is this study’s greatest weakness. Third, the study’s sample size was small, and certainly, a larger sample could have facilitated data evaluation. In fact, our cohorts might not have been sufficiently large for a meaningful pregnancy complication comparison. Each complication instance was infrequent as well, as Table 4 shows. The strength of this study, meanwhile, is its status as the largest cohort study to have evaluated postoperative adhesion and adverse pregnancy outcomes between BS and CS.

In conclusion, our data indicated that the incidence of postoperative adhesion after MIM with BS was similar to that with CS. Moreover, it seems that the suture type does not have a significant effect on pregnancy outcome.

## Figures and Tables

**Table 1:** Baseline characteristics of women at myomectomy.

		All			LM Only	
Characteristics	Conventional Suture (n = 94)	Barbed Suture (n = 97)	*p*	Conventional Suture (n = 93)	Barbed Suture (n = 44)	*p*
Age, year	32.9 ± 3.2	35.4 ± 3.2	<0.001	32.9 ± 3.2	35.6 ± 3.3	<0.001
BMI, kg/m^2^	21.4 ± 2.8	22.8 ± 3.5	0.003	21.4 ± 2.8	22.4 ± 3.9	0.08
Nulliparous			0.029			0.102
No	94 (100)	91 (93.8)		93 (100)	42 (95.5)	
Yes	0 (0)	6(6.2)		0 (0)	2(4.5)	
Abdominal surgery history			0.483			0.635
No	81 (86.2)	80 (82.5)		81 (87.1)	37 (84.1)	
Yes	13 (13.8)	17 (17.5)		12 (12.9)	7 (15.9)	
Preoperative symptoms			0.696			0.735
Bleeding	18 (19.1)	61 (16.5)		17 (18.3)	11 (25.0)	
Pain	15 (16.0)	11 (11.3)		15 (16.1)	4 (9.1)	
Compression	5 (5.3)	6 (6.2)		5 (5.4)	0 (0)	
Infertility	43 (45.7)	43 (44.3)		43 (46.2)	19 (43.2)	
Growing size	6 (6.4)	12 (12.4)		6 (6.5)	6 (13.6)	
No symptoms	7 (7.4)	9 (9.3)		7 (7.5)	4 (9.1)	
Adhesions at myomectomy			0.443			0.873
No	60 (63.8)	67 (69.1)		60 (64.5)	29 (65.9)	
Yes	34 (36.2)	30 (30.9)		33 (35.5)	15 (34.1)	
Type of operation			<0.001			-
Laparoscopy	93 (98.9)	44 (45.4)		93 (100)	44 (100)	
Robot	1 (1.1)	53 (54.6)		0	0	
Use of adhesion barrier			0.357			0.920
No	34 (36.2)	29 (29.9)		33 (35.5)	16 (36.4)	
Yes	60 (63.8)	68 (70.1)		60 (64.5)	28 (63.6)	
Operative time, min	126.9 ± 44.94	157.1 ± 59.5	<0.001	126.4 ± 44.89	129.6 ± 58.3	0.731
Estimated blood loss, mL	247.2 ± 215.0	267.9 ± 204.7	0.498	247.7 ± 216.1	247.7 ± 202.6	1.0
Concurrent ovarian surgery			0.383			0.492
No	61(64.9)	57 (58.8)		60 (64.5)	31 (70.5)	
Yes	33 (35.1)	407 (41.2)		33 (35.5)	13 (29.5)	
Transfusion			0.721			1.0
No	91 (96.8)	92 (94.8)		90 (96.8)	43 (97.7)	
Yes	3 (3.2)	5 (5.2)		3 (3.2)	1 (2.3)	
Endometrium exposure			0.797			0.291
No	89(94.7)	91 (93.8)		88 (94.6)	39 (88.6)	
Yes	5 (5.3)	6 (6.2)		5 (5.4)	5 (11.4)	
Total myoma, n	3.5 ±3.1	5.3 ± 4.6	0.001	3.5 ± 3.2	4.3 ± 4.0	0.117
Largest myoma						
Size, cm	5.8 ± 2.7	7.0 ± 2.2	0.001	5.8 ± 2.7	6.2 ± 1.9	0.289
Location			0.683			0.461
Anterior	40 (42.6)	40 (41.2)		40 (43.0)	22 (50.0)	
Fundus	39 (41.5)	35 (36.1)		38 (40.9)	15 (34.1)	
Posterior	9 (9.6)	13 (13.4)		9 (9.7)	6 (13.6)	
Lateral	6 (6.4)	9 (9.3)		6 (6.5)	1 (2.3)	
Type (FIGO classification)			0.654			0.066
Submucosal (1–2)	3 (3.2)	2 (2.1)		3 (3.2)	2 (4.5)	
Deep intramural (3–4)	47 (50.0)	50 (51.5)		46 (49.5)	27 (61.4)	
Intramural (5)	22 (23.4)	29 (29.9)		22 (23.7)	9 (20.5)	
Subserosal (6)	17 (18.1)	14 (14.4)		17 (18.3)	6 (13.6)	
Pedunculated (7)	1 (1.1)	1 (1.0)		1 (1.1)	0 (0)	
Intraligamentary (8)	4 (4.3)	1 (1.0)		4 (4.3)	0 (0)	
Tumor weight, g	127.2 ± 129.8	198.3 ± 185.7	0.003	125.9 ± 129.9	124.6 ± 99.1	0.955

Values are presented as number (%), median (range) or mean ± standard deviation. BMI, Body mass index; n, number; LM, laparoscopic myomectomy; RM, robotic myomectomy; FIGO, The International Federation of Gynecology and Obstetrics.

**Table 2:** Pregnancy outcomes after myomectomy.

		All			LM Only	
Parameter	Conventional Suture (n = 94)	Barbed Suture (n = 97)	*p*	Conventional Suture (n = 93)	Barbed Suture (n = 44)	*p*
			0.554			0.224
Term cesarean delivery	85 (90.5)	87 (89.7)		84 (90.3)	36 (81.8)	
Preterm cesarean delivery	3 (3.2)	6 (6.2)		3 (3.2)	4 (9.1)	
Miscarriage	5 (5.3)	4 (4.1)		5 (5.4)	4 (9.1)	
Hysterotomy due to IUFD	1 (1.1)	0 (0)		1 (1.1)	0 (0)	
Vaginal delivery	0 (0)	0 (0)		0 (0)	0 (0)	

Values are presented as number (%). LM, laparoscopic myomectomy; n, number.

**Table 3:** Surgical characteristics and outcomes at cesarean section.

		All			LM Only	
Parameter	Conventional Suture (n = 88)	Barbed Suture (n = 93)	*p*	Conventional Suture (n = 87)	Barbed Suture (n = 40)	*p*
Age (years)	36.5 ± 2.9	37.1 ± 3.1	0.120	36.4 ± 2.9	37.6 ± 3.3	0.037
Coexisting diseases						
Diabetes mellitus	9 (10.2)	8 (8.6)	0.708	9 (10.3)	1 (2.5)	0.169
Hypertension	4 (4.5)	4 (4.3)	1.0	4 (4.6)	2 (5.0)	1.0
Hypothyroidism	10 (11.4)	14 (15.1)	0.464	10 (11.5)	5 (12.5)	1.0
Thyrotoxicosis	3 (3.4)	3 (3.2)	1.0	3 (3.4)	1 (2.5)	1.0
Operative time (min)	57.4 ± 10.7	58.8 ± 11.7	0.120	57.3 ± 10.7	519.3 ± 10.5	0.332
Estimated blood loss (mL)	605.7 ± 210.8	563.8 ± 115.3	0.096	606.9 ± 211.7	572.5 ± 115.3	0.338
Presence of adhesion			0.095			0.153
No	48 (54.5)	62 (66.7)		47 (54.0)	27 (67.5)	
Yes	40 (45.5)	31 (43.7)		40 (46.0)	13 (32.5)	
Adhesion site			0.158			0.652
Bladder	4 (10.0)	8 (25.8)		4 (10.0)	3 (23.1)	
Omentum	2 (5.0)	2 (6.5)		2 (5.0)	1 (7.7)	
Adnexa	4 (10.0)	4 (12.9)		4 (10.0)	0 (0)	
Intestine	21 (52.5)	8 (25.8)		21 (52.5)	4 (30.8)	
Uterus alone	9 (22.5)	9 (29.0)		9 (22.5)	5 (38.5)	
Birth weight of baby (g)	3004.9 ± 415.0	3073.8 ± 508.7	0.320	3004.5 ± 417.4	2991.3 ± 611.2	0.887
Apgar score at 1 min	7.8 ± 0.9	7.9 ± 0.4	0.349	7.8 ± 0.9	7.9 ± 0.4	0.532
Apgar score at 5 min	8.8 ± 1.1	9.0 ± 0.1	0.078	8.8 ± 1.0	9.0 ± 0.2	0.114

Values are presented as number (%), median (range) or mean ± standard deviation. LM, laparoscopic myomectomy.

**Table 4:** Pregnancy complications in women at cesarean section.

		All			LM Only	
Parameter	Conventional Suture (n = 88)	Barbed Suture (n = 93)	*p*	Conventional Suture (n = 87)	Barbed Suture (n = 40)	*p*
Admission due to preterm labor	14 (15.9)	10 (10.6)	0.294	14 (16.1)	7 (17.1)	0.889
Pregnancy-induced hypertension	3 (3.4)	3 (3.3)	1.0	2 (2.3)	1 (2.5)	1.0
Premature rupture of membranes	1 (1.1)	2 (2.2)	1.0	1 (1.1)	1 (2.5)	0.529
Uterine rupture	1 (1.1)	0 (0)	0.486	1 (1.1)	0 (0)	0.486
Placenta previa	1 (1.1)	0 (0)	0.486	1 (1.1)	0 (0)	1.0
Placenta accrete	2 (2.3)	4 (4.3)	0.683	2 (2.3)	2 (5.0)	0.59
Placenta abruption	0 (0)	1 (1.1)	1.0	0 (0)	1 (2.5)	0.315

Values are presented as number (%). LM, laparoscopic myomectomy.

**Table 5:** Univariate and multivariate analysis for independent risk factors regarding adhesion formation after myomectomy.

	Univariate Analysis		Multivariate Analysis	
Clinical Factors	OR (95% CI)	*p*	OR (95% CI)	*p*
Age ≥ 34 ^a^	1.317 (0.625–2.776)	0.470		
BMI ≥ 22.1 kg/m^2 b^	0.809 (0.386–1.697)	0.576		
Type of operation (LM vs. RM)	0.707 (0.259–1.931)	0.707		
Suture methods (Conventional vs. Barbed)	0.561 (0.221–1.423)	0.224		
Operative time ≥ 142.3 min ^b^	8.207(3.330–20.228)	<0.001	0.132 (0.055–0.315)	<0.001
Multiple myoma (n ≥ 5 ^a^)	2.585(1.186–5.633)	0.017	0.470 (0.225–0.981)	0.044
Lst myoma ≥ 4.4 cm ^b^	0.331(0.124–0.887)	0.028	3.360 (1.287–8.769)	0.013
Sum weight ≥ 164.1 g ^b^	0.438 (0.182–1.052)	0.065	2.742 (1.170–6.427)	0.020

CI, confidence interval; vs., versus; OR, odds ratio; Lst, largest; LM, laparoscopic myomectomy; RM, Robotic myomectomy; ^a^ Median value in study population, ^b^ Mean value in study population.

## Data Availability

Not applicable.
